# Exploring the sensitivity of episodic and spatial memory tests to healthy and pathological cognitive aging

**DOI:** 10.3389/fnagi.2025.1547834

**Published:** 2025-06-20

**Authors:** Gina Michallat-Bragg, Megan Bennett, Bethany Iona Flewitt, Sayed Kazmi, Sarah Jane Smith, Christine Wells, Annabel Hollins, Charlotte Ash, Sarah Thwaites, Wendy Neil, David Howett, Sarah Dexter-Smith, Dennis Chan, James Dachtler, Steven Poulter, Stephen Evans, Colin Lever

**Affiliations:** ^1^Department of Psychology, Durham University, Durham, United Kingdom; ^2^Ascentys Limited, Bradford, United Kingdom; ^3^Centre for Dementia Research, Leeds Beckett University, Leeds, United Kingdom; ^4^School of Social and Health Sciences, Leeds Trinity University, Leeds, United Kingdom; ^5^Cumbria Partnership NHS Foundation Trust, School of Psychology, Newcastle University, Newcastle, United Kingdom; ^6^Leeds and York Partnership NHS Trust, Leeds, United Kingdom; ^7^School of Psychological Science, University of Bristol, Bristol, United Kingdom; ^8^Tees, Esk and Wear Valleys NHS Foundation Trust, Darlington, United Kingdom; ^9^Institute of Cognitive Neuroscience, University College London, London, United Kingdom; ^10^The Department of Psychological Medicine, The Old Chapel, Bootham Park, York and Scarborough Teaching Hospitals NHS Foundation Trust, York, United Kingdom

**Keywords:** hippocampus, Alzheimer’s disease, cognitive aging, aging, spatial memory, sequence memory, episodic memory

## Abstract

**Introduction:**

In an increasingly aging society, testing hippocampal-dependent cognition in a quick and low resource manner will be crucial in: assessing the potential benefits of lifestyle choices and interventions affecting cognitive ageing (such as those involving exercise, diet, and sleep); detecting pathological aging, such as in Alzheimer’s disease, where hippocampal degeneration occurs relatively early on.

**Methods:**

Over 300 participants aged 18-89 completed three cognitive tests, namely the Addenbrooke’s Cognitive Examination-III (ACE-III), The Four Mountains Task (4MT), and a new task introduced here, the Spaces and Sequences Episodic Video Task (SSEVT). Hippocampal tissue is particularly vulnerable to aging, and the 4MT and SSEVT were designed to be hippocampal-dependent. Accordingly, we tested the hypothesis that 4MT and SSEVT performance would be significantly compromised by aging. As an initial proof-of-concept exploration of these tests’ ability to detect pathological aging, such as in Alzheimer’s disease, we compared 10 patients with Mild Cognitive Impairment (MCI) with matched subsamples of the older group (Healthy ageing, HA).

**Results:**

Supporting the hippocampal-aging related hypothesis, 4MT and SSEVT scores showed appreciably stronger age-related declines than ACE-III scores. The middle-aged group (mean: ∼51 years) were significantly worse than the young group (mean: ∼21 years) on the 4MT (Cohen’s d = 0.724) and the SSEVT (Cohen’s d = 0.443); and the older group (mean: ∼71 years) were significantly worse than the middle-aged group on the SSEVT (Cohen’s d = 0.724). Neither pattern was seen for ACE-III. Suggestively, the MCI patients performed worse than the matched HA group on the 4MT (consistent with previous work), and on our novel SSEVT, but not on the ACE-III.

**Discussion:**

We conclude that the 4MT and SSEVT may be suitable for assessing lifestyle choices and interventions affecting cognitive ageing. We also propose that these findings provide an initial proof-of-concept for these tests’ ability to detect pathological aging in its early stages and support further exploration of this with larger clinical samples.

## 1 Introduction

### 1.1 Need for early diagnosis of Alzheimer’s disease (AD)

Alzheimer’s disease (AD) is a neurodegenerative disorder characterized by progressive neuronal death (Braak and Braak, 1995; Hardy and Higgins, 1992). If identified in its prodromal stages, AD progression may be decelerated via protective lifestyle factors (Frisoni et al., 2020) such as social embeddedness and exercise (Cass, 2017; Edwards et al., 2019; Norton et al., 2014) and the emergence of anti-amyloid immunotherapies such as lecanemab raises the possibility of future pharmacological disease modification or potentially, even pharmacological treatments (Aisen et al., 2017; Sims et al., 2023; Van Dyck et al., 2023). However, for any such treatments to be effective, they must be targeted to those in the early stages of the disease. In these prodromal stages, AD present clinically as mild cognitive impairment (MCI; Albert et al., 2011), in which there is evidence of subjective and objective cognitive impairment but with intact daily living activities, but on clinical grounds alone MCI due to AD can be hard to distinguish from other causes of MCI such as anxiety. The forecasted near tripling of global AD cases from 2019 to 2050 (Nichols and Vos, 2021), currently estimated as ∼35% of over-85’s (Alzheimer’s Association, 2021), reinforces the pressing need for new screening measures able to identify the disease early.

### 1.2 Using hippocampal-dependent tasks to assess early stages of Alzheimer’s disease (AD) and healthy cognitive aging

Though AD pathology is widespread throughout the neocortex in its later stages, the early stages of AD is characterized by changes localized to the hippocampal formation, and to the rhinal cortices which provide input to the hippocampus (Braak et al., 1993; Gómez-Isla et al., 1996; Wang et al., 2003; Whitwell et al., 2007; Braak and Del Tredici, 2015). While such post-mortem staging evidence has been taken to suggest an obligatorily rhinal first stage, more recent evidence suggests hippocampal as well as rhinal initial staging posts of neurodegeneration (Ravikumar et al., 2024). As such, detection of hippocampal damage may aid detection of early AD, increasing diagnostic confidence when combined with blood biomarkers of β-amyloid and tau pathology that will be available for clinical use in the near future. However, measurement of hippocampal atrophy using structural brain imaging has been found on systematic review to have low sensitivity and specificity for MCI due to AD and is therefore not recommended as a standalone add-on diagnostic test (Lombardi et al., 2020).

Tests of hippocampal-dependent cognition may provide some benefit here. While neuropsychological cognitive tests cannot of course replace biomarker tests and clinical assessments, they may have some advantages as initial or additional screening tools (e.g., non-invasive, easier to upscale). In particular, there is likely strong benefit to short cognitive tests which can provide complementary diagnostic information, potentially increasing the sensitivity and, crucially, specificity afforded by current tests (Laske et al., 2015; Wood et al., 2016). More complex test batteries could be employed at later stages for specifically at-risk categories. Tasks targeting dysfunction in the Entorhinal cortex could also be promising, (e.g., Howett et al., 2019) but remain at present in the research domain and are yet to undergo the same degree of validation and large-sample testing as hippocampal tests.

Given the size of the aging population, cognitive tests of hippocampal function that can be applied at scale and at low cost and user burden will be of great benefit in assessing the potential benefits of lifestyle choices and pro-cognitive interventions such as those involving sleep, diet, stress-reduction, and exercise (e.g., Meerlo et al., 2009; Erickson et al., 2011; Duzel et al., 2016; Fjell et al., 2020). The hippocampus is one of very few brain regions where neurogenesis occurs in the adult human, and thus likely has a privileged role in mediating healthy cognition in aging. Taking exercise as an example, cross-sectional and longitudinal evidence indicates that cardiorespiratory-focused exercise boosts hippocampal volume, and spatial memory (Erickson et al., 2011; Aghjayan et al., 2021), effects at least partly dependent upon neurogenesis (Van Praag, 2008; Erickson et al., 2011; Liu and Nusslock, 2018).

In summary, hippocampal-dependent cognitive tests could therefore be useful not just as screening tools for early AD, but in exploring the nature of healthy cognitive aging and factors that affect it.

### 1.3 Hippocampal-dependent cognition: spatial and episodic memory

What cognitive functions depend upon the hippocampus? Many lines of evidence (e.g., reviewed O’Keefe and Nadel, 1978; Poulter et al., 2018) indicate an important role for the hippocampus in spatial memory. Briefly, these include: the discovery of cells in the hippocampal formation with spatial correlates (O’Keefe and Dostrovsky, 1971; Taube et al., 1990; Ekstrom et al., 2003; Hafting et al., 2005; Lever et al., 2009; Jacobs et al., 2013; Høydal et al., 2019; Poulter et al., 2021); impairments in spatial memory following hippocampal lesions (Broadbent et al., 2004; Kessels et al., 2001; Morris et al., 1982); and positive associations between spatial abilities on the one hand, and hippocampal volume (Schinazi et al., 2013; Woollett and Maguire, 2011) and activity (Burgess et al., 2002; Cornwell et al., 2008; Li and King, 2019) on the other.

Spatial memory likely shares underlying processes with episodic memory (e.g., O’Keefe and Nadel, 1978; Bird and Burgess, 2008; Buzsáki and Moser, 2013), and hippocampal-lesioned patients show a combination of spatial, episodic, and temporal memory deficits (Scoville and Milner, 1957; Vargha-Khadem et al., 1997). The latter may be linked to a hippocampal role in supporting memories for sequences of events (Fortin et al., 2002; Gilbert and Kesner, 2002; Kumaran and Maguire, 2006). Importantly, the hippocampus is thought crucial to episodic recollection, whilst other regions likely subserve familiarity-based recognition (Brown and Aggleton, 2001; Ranganath et al., 2004; Diana et al., 2007). Thus, a putative hippocampal-dependent episodic memory task should minimize contributions from purely familiarity-based recognition. This was an important consideration in the design of our novel “Spaces and Sequences Episodic Video task” (SSEVT) described below (see section “2.2 Tests and questionnaires”) and introduced in this report.

In addition to the lines of evidence outlined above, the fact that spatial and episodic memory are hippocampal-dependent aligns with the observed decline in hippocampal volume during healthy aging (Cansino, 2009; Heo et al., 2010). It is also consistent with the prominence of spatial and episodic memory deficits, contrasted with the relative preservation of semantic memory, in MCI and early stages of AD (Bäckman et al., 2001; Ghoshal et al., 2002; Greenaway et al., 2006; Grober et al., 2008; Di Paola et al., 2007; Lithfous et al., 2013; Mortamais et al., 2017; Murphy et al., 2008; Twamley et al., 2006).

### 1.4 Some limitations of existing screening tools for AD and cognitive aging

Despite the potential advantages of neuropsychological cognitive tests, the value of existing measures is constrained by several limitations. The most used tests of dementia in the United Kingdom are the Mini Mental State Examination (MMSE; Folstein et al., 1975), the Montreal Cognitive Assessment (Nasreddine et al., 2005), and Addenbrooke’s Cognitive Examination-III (ACE-III; Hsieh et al., 2013), all three being brief screens of global cognition. To date, evidence for the accuracy of these screens in identifying AD and MCI has been mixed. For example, some studies have found the ACE-III has high diagnostic accuracy in identifying mild AD (Matias-Guiu et al., 2017) and can differentiate between controls, MCI patients, and dementia patients (Potts et al., 2022). Comparatively, Beishon et al. (2019) systematic review concluded that there was insufficient evidence to recommend the use of ACE-III in screening for dementia or MCI in those at risk of cognitive decline, reporting variable sensitivity (dementia: 82%–97%; MCI: 75%–77%) and specificity (dementia: 4%–77%; MCI: 89%–92%). The latter also noted that no studies had been conducted in the primary care setting. Similarly, the accuracy of MMSE scores in the conversion of MCI to AD dementia has been shown to vary in sensitivity (27%–89%) and specificity (32%–90%, Arevalo-Rodriguez et al., 2015).

These screening tools are also subject to educational bias (Bruno and Schurmann Vignaga, 2019; Pan et al., 2022), influencing their accuracy of dementia diagnosis (Jubb and Evans, 2015). This may be because education provides a degree of “cognitive reserve” whereby a more-educated individual performs better in neuropsychological tests than a lesser educated individual, despite having the same degree of AD pathology (Wilson et al., 2019; Swanwick et al., 1999). Higher education seems to facilitate better performance on cognitive tests (Glymour et al., 2012) and a lower dementia risk (Caamaño-Isorna et al., 2006; Sharp and Gatz, 2011), consistent with cognitive reserve theory (Stern, 2012). However, some have found that higher education accelerates deterioration in AD once diagnosed (Scarmeas et al., 2006; Wilson et al., 2004) and does not affect rates of cognitive decline in healthily aging individuals (Lenehan et al., 2015). Regarding education, we suggest it is important, if difficult, to distinguish between two hypothetical factors: education as a factor conferring expertise advantages (e.g., re instructions, strategies, verbal associations) specific to a given neuropsychological task; and education as having provided a kind of directly neurotrophic factor which boosts real-world episodic memory in a more general manner dependent on healthier neural tissue. In our view, episodic memory test performance should ideally be dependent only upon this second factor.

In tasks such as the ACE-III, not only education but also verbal intelligence, proficiency in the test language, and cultural biases can modulate scores, potentially causing inequity in diagnostic provision across differing ethnocultural communities (Brown et al., 2021). These can be partially addressed by generating versions specific to a language/culture (Pan et al., 2022), but these may also run into difficulties (e.g., intersectional participants).

These existing measures also heavily rely on verbal tasks, which can be problematic when exploring medial temporal lobe functioning. For example, such tasks have limited utility in the context of patients with expressive aphasia. Visual tasks, by comparison, would not be limited by this issue. Furthermore, making tasks more reliant on visual than verbal elements has the potential to reduce the effect of education and sex on test performance (Kawano et al., 2010; Vadikolias et al., 2012) and to tap into dysfunction in the linguistically non-dominant hemisphere.

Few tests are currently available that are both suitable for application to clinical practice and can target episodic memory in an ecologically valid manner (Plancher et al., 2012). Current screening measures often rely on tasks that are not reflective of daily life and therefore may not be generalizable. Assessments that are reflective of genuine, real-life cognitive abilities are essential if we are to detect cognitive decline early, as these represent the clinical, functional impact of such cognitive changes (Bhargava et al., 2024). The most common episodic memory tests test verbal learning of word lists (e.g., RAVLT), and typically require specialized training to execute and score, as do most neuropsychological tests. This could be problematic if such tests are to be applied as screening tools on a large scale, bringing additional time and cost. Similarly, where there is possible variation in administration and interpreting scores, an element of bias and subjectivity is introduced. More importantly, verbal learning scores are often affected by secondary factors, including sex (Bolla-Wilson and Bleecker, 1986; Herlitz and Rehnman, 2008; Sundermann et al., 2019), verbal intelligence (Bolla-Wilson and Bleecker, 1986), proficiency in the test language (Blumenau and Broom, 2011), and education (Olaya et al., 2017; Van der Elst et al., 2005; Lou et al., 2022).

### 1.5 Could tests tapping the hippocampus fill the gap?

The above considerations suggest a need for tasks which test spatial and/or episodic memory in a hippocampal-dependent manner, while being minimally reliant on culture-specific knowledge and education-based expertise. Toward this aim, we explored the Four Mountains Test (4MT) and our novel “Spaces and Sequences Episodic Video Task” (SSEVT). The 4MT is a test of short-term, allocentric spatial memory, in which hippocampal-damaged patients show clear deficits (Hartley et al., 2007). 4MT scores correlate with hippocampal volume in healthy young adults (Hartley and Harlow, 2012). Regarding AD, 4MT scores have been shown to correlate with AD biomarkers in MCI patients (Moodley et al., 2015), predict MCI-to-AD conversion (Wood et al., 2016), and distinguish AD from Frontotemporal and Semantic Dementia (Bird et al., 2010; Pengas et al., 2010).

Our intention with the SSEVT (Figures 2, 3) was that it should be an ecologically-valid task, that it should be focused on hippocampus-dependent cognitions notably spatial and episodic memory, that it should be easy to administer, and ideally minimally dependent upon education-based expertise. See section “2.2.2 Spaces and sequences episodic video task (SSEVT)” below for further details regarding task design rationale, and specificity of each of the five types of questions. Briefly, the video in the SSEVT simulates a participant touring through a house from a first-person perspective. The tour lasts 256 s, and involves various episodic events taking place within eight rooms, with two visits to each of the eight rooms, and exposure to seven characters overall. Two characters appear three times, the others appear twice. The purpose of doubling exposures to characters and rooms is to generate interference, and bias successful strategies toward potentially hippocampus-reliant processes involving representations of context and sequences. Importantly, unlike most verbal tests of episodic memory, with a view to ease of administration, the SSEVT uses a forced-choice question format. After a 20 min interval, participants answer five sections of questions, each beginning with a practice question (Figure 2 shows example practice questions). Section 1 of the SSEVT examines character-event associations, section 2 examines object-context associations, and sections 3–5 all examine sequence memory, each with varying reliance on spatial context.

### 1.6 Study rationale and aims: key points

Evidence clearly shows (see section “1.3 Hippocampal-dependent cognition: spatial and episodic memory”) that hippocampal tissue integrity (e.g., volume) declines in aging. Consistent with this, hippocampal-dependent function such as episodic memory declines with aging, while other abilities such as semantic memory are relatively preserved, (e.g., Levine et al., 2002; Piolino et al., 2002). Both the 4MT and SSEVT were designed to be hippocampal-dependent (already shown for 4MT, see section “1.5 Could tests tapping the hippocampus fill the gap?”). Accordingly, to be useful as sensitive tests of hippocampal-dependent aging (while also being simple to administer and score), and thus potentially suitable for assessing the factors that modulate hippocampal-dependent aging, we reasoned that performance on the 4MT and SSEVT should show a clear decline with age. The first part of this study examined this prediction, while also exploring other potential modulators of performance on these tests, such as sex and education (see section “1.4 Some limitations of existing screening tools for AD and cognitive aging”).

The 4MT and SSEVT were also designed with a view to detecting early stages of AD. As an initial proof-of-concept test of this idea, in the second part of this study, we examined MCI-vs-Healthy Aging (HA) discrimination in three cognitive tests: the 4MT, SSEVT, and ACE-III. We compared performance of a small sample (*n* = 10) of MCI patients, diagnosed according to the Albert et al. (2011) criteria, against age-matched and education-matched healthy aging controls. Thus the second part of the study tested the prediction that 4MT and SSEVT would discriminate the MCI vs. HA groups.

## 2 Materials and methods

### 2.1 Participants

#### 2.1.1 Healthy sample

A total of 376 healthy participants (161 male, 215 female) aged 18–89 years-old (M = 42.26, SD = 22.56) were recruited from the general population via opportunistic sampling, including through the Durham University Participant Pool and advertisements on social media. Racial identity was largely White British/Irish (matching the MCI sample) as follows. White (including British/Irish/European/other White), *n* = 249; Black (various), *n* = 7; Asian (including East Asian and South Asian) *n* = 21; Mixed, *n* = 33; Other/data not given/available, *n* = 66. Exclusion criteria were as follows: (1) Presence of significant neurological conditions; (2) Major psychiatric disorders; (3) The use of cognitive enhancing drugs e.g., Cholinesterase Inhibitors; (4) A history of alcohol excess or illicit drug use resulting in functional issues or reliance on external assistance; (5) The diagnosis of any form of dementia; (6) Individuals under the age of 18. All participants were required to be fluent in English.

#### 2.1.2 Cognitively impaired sample

A total of 10 MCI patients (five male, five female) aged 62–86 (M = 73.30, SD = 8.47), all White British, took part in the study. All had received a diagnosis of MCI, according to the Albert et al. (2011) criteria, within the previous 3 months: this time frame was adopted to ensure that patients’ cognition had not appreciably declined further in the interim between diagnosis and testing. Participants were recruited from four different sites: Tees Esk and Wear Valleys NHS Foundation Trust (*N* = 1); Cumbria Partnership NHS Foundation Trust (*N* = 5); Leeds and York Partnership NHS Foundation Trust (*N* = 3); and South Tees Hospitals NHS Foundation Trust (*N* = 1). Exclusion criteria were as above for healthy participants. Ethical approval for this research was given by the United Kingdom Health Research Authority and the Durham University Ethics Committee.

### 2.2 Tests and questionnaires

#### 2.2.1 Four Mountains Task

**FIGURE 1:**
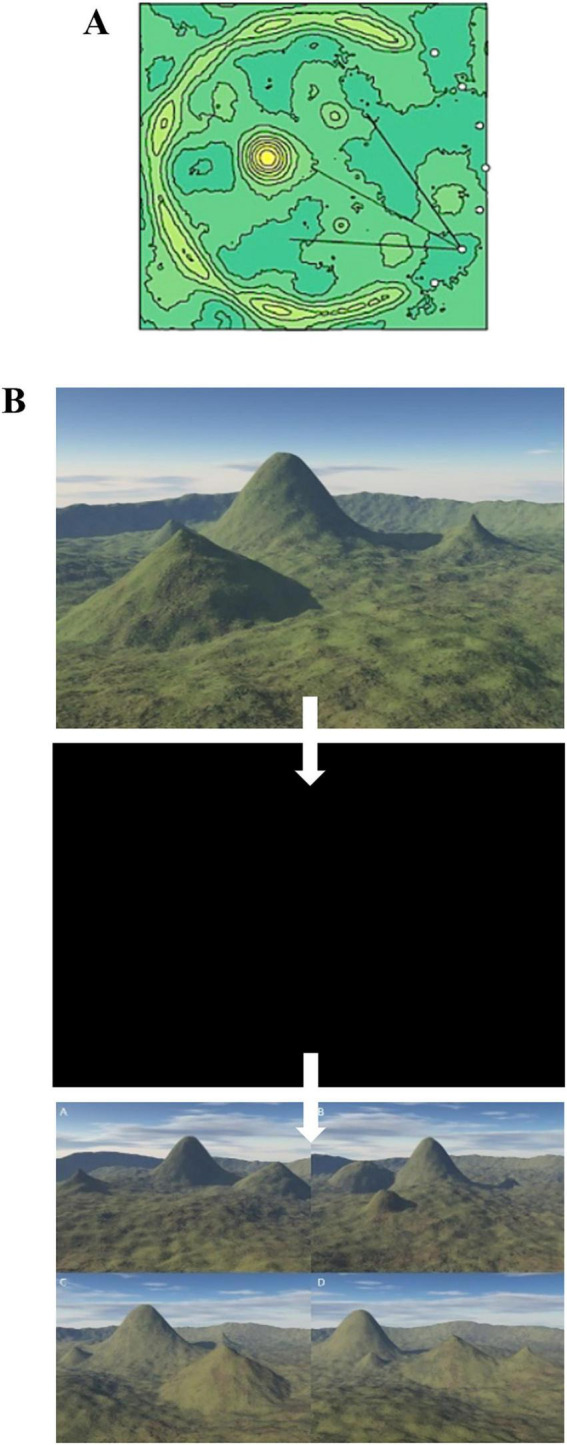
The Four Mountains Test. Stimuli used for the Four Mountains Test. **(A)** Each landscape consists of four hills of varying shape and size in a semicircular range. Each stimulus used a unique configuration of hills in addition to various combinations of non-spatial parameters, including cloud cover, lighting, texture, and vegetation color, to create naturalistic variation in the scenes. The scenes are shown as though through a virtual camera sited at one of seven viewpoints (shown by white circles), each equidistant from the center of the circular field. **(B)** Example of one trial within the test. The subject is first presented with a rendered stimulus image based on the topography described above in panel **(A)**. They are then presented with four scenes comprising varying topographical landscapes. One of these scenes depicts the same topographical landscape as shown in the previous scene but viewed from a different angle (one of the seven pre- determined viewpoints). The other three images show different scenes, where the size, shape, or layout of the mountains have changed. The correct answer is the image showing the same topographical landscape. Participants complete fifteen of these trials.

The 4MT is a test of working allocentric memory that assesses the participants’ ability to recall the spatial configuration of a series of computer-generated landscapes from different viewpoints; this is designed to reflect the role of the hippocampus in spatial cognition. The rationale behind the 4MT has two key foundations: (1) The hippocampus is affected early in AD pathology [see section “1.2 Using hippocampal-dependent tasks to assess early stages of Alzheimer’s disease (AD) and healthy cognitive aging” above]; (2) The hippocampus is critical in spatial memory (see section “1.3 Hippocampal-dependent cognition: spatial and episodic memory”). A test such as the 4MT, which explores allocentric, view-independent spatial memory, should be highly dependent on the integrity of hippocampal tissue, and thus sensitive to the damage caused by early stages of AD. Participants complete 15 trials in which they are first shown a landscape depicting four mountains, and then after a two second delay, have to identify which of four images shows the same initial landscape, but viewed from a different perspective. For the full procedure of the 4MT, (see Chan et al., 2016; Hartley et al., 2007; Bird et al., 2010; Moodley et al., 2015). Figure 1A below shows a plan view of the computer-generated landscape, with the seven possible viewpoints shown, whilst Figure 1B shows an example trial. Participants recorded their answers on an A4 answer sheet.

**FIGURE 2:**
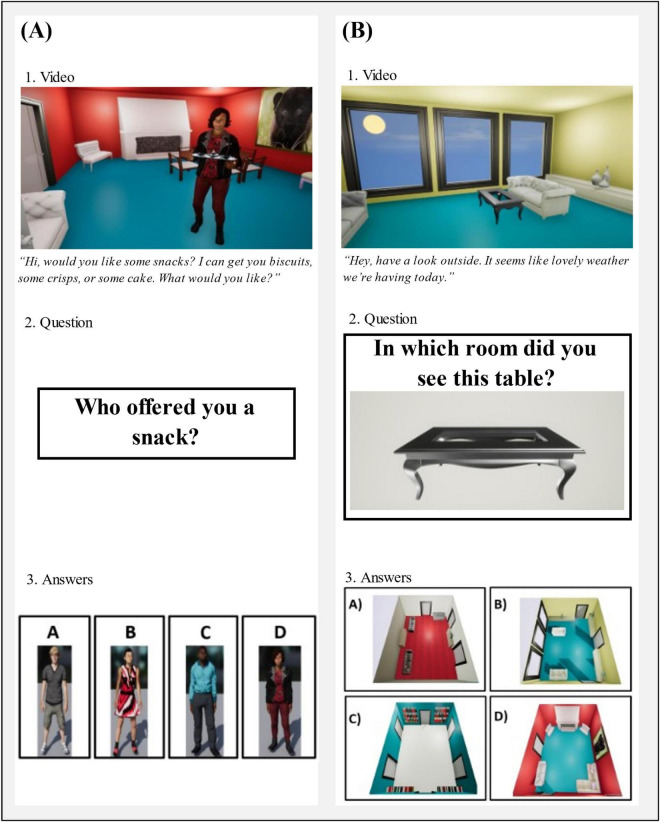
Example practice questions from sections 1 to 2 of the Spatial and Sequence Episodic Video Task (SSEVT). During the video, participants see multiple rooms, characters, objects, and events. After a retention interval, the participant is then asked 20 multiple-choice questions about what they saw, divided into five sections. **(A)** An example from section 1 of the video: character questions. During the video, participants see a woman holding a tray who offers them a variety of snacks. During the questionnaire stage, they are then asked, “Who offered you a snack?”. The participant is shown four options of different characters, one of whom being the correct character seen in the video. The other three characters all appeared at some point in the video and were set against neutral backgrounds, preventing recall by familiarity alone. **(B)** An example of a question from section 2 (object-based questions). During the video, the participant sees a woman looking out the window of a room with blue flooring, yellow walls, and various pieces of furniture, including a black coffee table. They are later asked, “In which room did you see this table?”. The participant is then shown four different options of rooms, one of which being the correct room as seen in the video. The three other rooms contain features present in then video so that the correct answer cannot be identified by familiarity alone.

**FIGURE 3:**
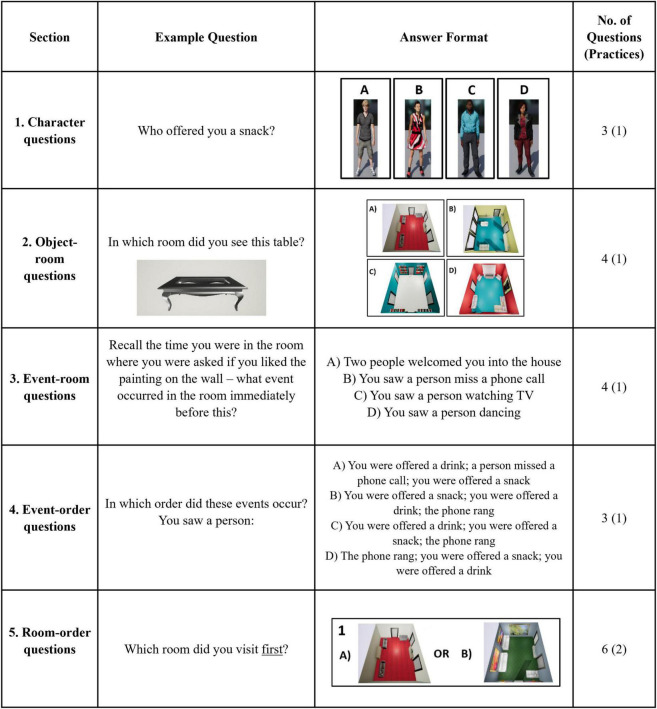
The practice questions for each of the five sections of the SSEVT. The SSEVT has five sections as shown in the Figure above. Sections focus upon; Person associations, object location, Event order (Immediately before/after), Event order (Multiple events), and Room order. Each section was preceded by a practice question. Responses were forced choice with one correct answer and either three (see sections “1 Introduction”–“4 Discussion”) or one (section 5) other option(s) as lures. No feedback was given for either practice or scored questions.

#### 2.2.2 Spaces and Sequences Episodic Video Task (SSEVT)

Key design aims of this task included the following. We aimed to create an ecologically valid first-person perspective task assessing spatial and episodic recall that was easy to administer. To render scoring expertise unnecessary, we implemented a forced-choice question format. With a view to the putative hippocampal-dependence of the task, we sought to minimize the ability of familiarity-dependent recognition processes to task success. For example: (a) each of the eight rooms is visited twice, with a different event on each visit; (b) person-association questions presented characters who all appeared within the video and were therefore all familiar, with character images set against neutral backgrounds to minimize snapshot memory contributions; (c) object-room association questions presented objects against neutral backgrounds, and bird’s eye views of rooms that were not previously presented in the video itself. With these aims in mind, the SSEVT (see Supplementary Materials 1) was created by authors CL, SK, JD, CW, SS, and GM-B. The SSEVT simulates a participant touring through a house from a first-person perspective. During the 256 s video, the individual moves through a house made up of eight distinctly decorated rooms. Across these rooms the viewer is exposed to seven people (four women and three men). Two characters appear three times, whilst all others appear twice. Within each room, a particular event occurs, such as someone watching TV, talking about the weather, or hanging up a picture (see Figure 2 for example screenshots of the video). Before the video begins, the participant is presented with the following script: “The video you are about to watch is presented as if you were walking through a computerized house. The video lasts approximately 4.5 min and various things will happen as you walk through the house. Please pay attention to the video and remember what you can as you will be asked questions about the video 20 min after it finishes. The questions will assess how well you can remember; the people, the objects, the rooms, and the events.” A total of 20 min after viewing the video, the participant answers 20 forced-choice questions over five sections. Each section began with a practice question to familiarize the participant with the up-coming question format. Two practice questions are shown as examples in Figure 2.

For a visual overview and example questions for each section, see Figure 3. Section 1 assesses character-based associations. These questions require the participant to identify which of four possible individuals (all of whom appeared in the video) were involved in a given event. In Section 2, participants are shown an object and are asked in which room it appeared. This aims to tap into hippocampal functioning by assessing associations between a spatial context and its contents (object-room associations). Sections 3–5 all examine sequence memory, but each with a different reliance on spatial context. In Section 3, participants are reminded of an event that occurred (e.g., they saw a person watching TV) and are asked to recall what happened in the room immediately before it. This therefore relies on participants being able to recall the order (sequence) of events. Comparatively, Section 4 presents participants with three separate events and asks them to place them in the correct order that they occurred in the video. This again relies on memory of event order but does not link events to rooms. Finally, section 5 does not necessitate memory of events, but instead asks participants to recall the order in which they visited different rooms. Participants are shown pictures of two different rooms and asked, “Which room did you visit first” or “Which room did you visit most recently?”. For Sections 1-4, participants had four options to choose from, and each correct answer scored one point. For section 5, as they had only two options to choose from, each correct answer scored 0.5 points. The total score on the SSEVT was the sum of points from all five sections. Accordingly, with six questions in section 5, the maximum score on the SSEVT was 17 points.

#### 2.2.3 Addenbrooke’s Cognitive Examination III (ACE-III)

The ACE-III is a screening tool for global cognition that is free to use and has been widely adopted in the United Kingdom’s NHS, and elsewhere (Hsieh et al., 2013). Due to this widespread use, the ACE-III was included in the testing battery of this project. The test is paper-based and includes items assessing attention, memory, fluency, language, and visuospatial abilities, with a maximum score of 100.

#### 2.2.4 Spatial Abilities and Practices Questionnaire (SAPQ)

The rationale for the need for hippocampal-dependent tasks for assessing healthy aging and early stages of AD also apply to questionnaires. With this in mind, the SAPQ (Supplementary Materials 1) was developed by authors CL, GM-B, DC, and DH to assess spatial memory and navigational ability in both familiar and unfamiliar environments in the real world, and is made freely-available here. The questionnaire consists of 15 questions assessing different kinds of spatial ability, in the form of statements to endorse using a Likert scale, e.g., “I have difficulty accurately visualizing in my mind’s eye the local walking routes (e.g., to shops, pubs, parks, restaurants) to and from my home.” One important aim in designing the SAPQ was to minimize potential misunderstanding of terminology and the need for abstract reflection by including specific questions that may be friendlier to answer for cognitively aging participants. Thus, the questions often contain examples, such as “when I walk out of a large shop or shopping center,” and “local walking routes (e.g., to shops, pubs, parks, restaurants),” designed to try to jog typical memories and knowledge of habits from the participant’s mind. Another aim was to capture potential decline in spatial abilities. Accordingly, every question in the SAPQ is followed by the question “Has this ability changed within the last 12 months?”, to gauge potential decline. This was scored from −2 to +2 (indicating direction of change) and then added or deducted from the original score. One potential drawback associated with the use of self-report questionnaires is the issue of illness insight, relevant of course for MCI and AD, as insight into the disease declines as it progresses (Migliorelli et al., 1995; Starkstein et al., 1996). This kind of anosognosia (Horning et al., 2014) can affect the way in which disease-affected individuals answer questionnaires (Bédard et al., 2003; Farias et al., 2005), such as those tapping spatial navigation abilities: participants may overestimate their navigational abilities. In order to mitigate this in the present study, we allowed participants to get help (from a partner, family member, or other career) in answering questions, with a tick box to specify if the participant answered the questions alone or with assistance.

#### 2.2.5 Social Networks and Social Embeddedness Questionnaire (SNSEQ)

Having rich and meaningful social networks (being “socially embedded”) appears to protect against cognitive decline (Giles et al., 2012; Holtzman et al., 2004). Importantly, this may be achieved through hippocampal modulation (Branchi et al., 2006; Smith et al., 2018). Larger social networks are thought to provide a form of “social buffering,” which protects against AD pathology (Davitz and Mason, 1955; Coe et al., 1978). Those with smaller social networks may therefore have a greater dementia risk (Bennett et al., 2006; Fratiglioni et al., 2004). Consequently, measuring social embeddedness may be important in understanding AD risk. With this in mind, this study introduces the SNSEQ (Supplementary Materials 2) as a freely-available tool in the assessment of healthy and pathological aging. The SNSEQ was developed by authors GM-B, JD, and CL. It consists of 16 questions in the form of statements to endorse using a Likert scale [(1). Strongly disagree, (2). Disagree, (3). Neither agree not disagree, (4). Agree, or (5). Strongly agree]. As with the SAPQ, each item on the SNSEQ incorporates a measure of change: a standard Likert-scale question which asks whether there has been a change in the last 12 months and in which direction. It includes assessment of social motivation, and reliance on others to initiate and maintain social exchanges, as well as the number and quality of social networks. As with the SAPQ (see above), participants could receive help in answering questions, with a tick box to indicate if such assistance was used.

#### 2.2.6 UCLA Loneliness Scale (ULS) and Satisfaction with Life Scale (SWLS)

Evidence for a relationship between loneliness and cognitive-aging has so-far proved inconclusive (Lara et al., 2019a,2019b; Victor, 2021) but has been associated with hippocampal volume (Düzel et al., 2019) and a heightened AD-dementia risk (Sundström et al., 2020). Life satisfaction may also influence cognition (Llewellyn et al., 2008; Goveas et al., 2016). Life satisfaction was measured using the Satisfaction with Life Scale (SWLS; Diener et al., 1985) and subjective loneliness was measured using the UCLA Loneliness Scale (ULS; Russell et al., 1978).

### 2.3 Procedure

#### 2.3.1 Healthy controls

The cognitive tasks were tested in the sequence: 4MT first, SSEVT second, and ACE-III last. Testing was conducted in a quiet environment (Durham University’s Psychology Department, private homes, and occasionally quiet public places e.g., church hall). To explore the relationship between age and test performance, for some analyses the sample was divided into three groups: Young, 18–35; Middle-aged, 36–61; and Older people, 62–89.

#### 2.3.2 MCI patients

Patients were diagnosed as having MCI according to the (Albert et al., 2011) criteria, by one of the four NHS trusts (see section “2.1 Participants” above). To have been diagnosed with MCI, participants had already taken several cognitive tests. A few patients had undergone biomarker testing, but this varied across the NHS trusts, and the sample n would be too small for meaningful analysis. Discussions with the referring site established which tests included in the project battery each patient had already completed, to prevent unnecessary duplication and/or practice effects. Potential participants were provided with an information sheet for the project, with a number to call if they were interested in participating. A follow-up letter was then sent to their address. Before testing began, informed consent was sought from the patient for use of their data, comprising: (1) their previous clinical data, notably the results from the NHS site-specific psychological test and questionnaire battery; (2) results from our additional tests; (3) any diagnostic changes made at a future time. The project-specific battery consisted of the following tasks in the following order, although for many the ACE-III had already been administered so was not repeated: 4MT; SSEVT; TMT-B; SNSEQ; RAVLT; SAPQ; and the ACE-III. Testing was conducted either at an NHS clinic (*n* = 1) or at the participant’s home at their request (*n* = 9), taking care to implement safety guidelines in accordance with risk assessments.

### 2.4 Data analysis

Relationships between variables in the healthy sample were explored using both Pearson and Spearman’s correlations, with Welch ANOVAs with Games Howell *post hoc* tests then probing differences between age-demographics. Welch tests were used to test for differences in sex and education within the sample. Multiple regressions were used to probe whether age could predict cognitive performance, with sex and education included as covariates. Differences between MCI patients and controls were probed using Welch tests. Welch tests were used as they are generally considered more robust than student *t*-tests or one-way ANOVAs. Participants with a missing value(s) for a measure were simply excluded from that measure whist retaining their other scores. A maximum of 87 participants per measure were excluded in this way (minimum sample size 289). For the analysis presented in Results below, 12 participants’ ACE-III scores were removed; their scores were low outliers for their age-groups (young, middle-aged, old), presumably attributable to imperfect application of the English fluency requirement. Analysis including these 12 datapoints affects none of the results reported below, namely: (a) ACE-III had a much lower sensitivity to age (all included: β = −0.132; below: β = −0.126) than 4MT and SSEVT; (b) education positively predicted ACE-III scores (all included: β = 0.249; below: β = 0.212), more so than 4MT and SSEVT; (c) ACE-III performance did not discriminate MCI patients from Healthy-aging controls (Welch t test all included: *p* = 0.466; below: *p* = 0.348).

## 3 Results

### 3.1 Overview: healthy participant characteristics and sex differences

**FIGURE 4:**
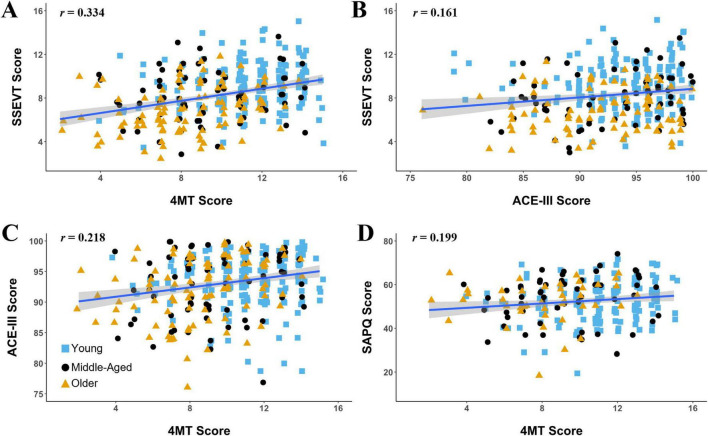
Different between-task correlations for the three cognitive tasks: the Four Mountains task (4MT), the Spaces and Sequences Episodic Video task (SSEVT), and Addenbrooke’s Cognitive Examination, version 3 (ACE-III). Between-task correlations for the three cognitive tasks. The highest correlation (r = 0.334) was seen for scores on the two tasks designed to be hippocampus dependent: the 4MT and SSEVT **(A)**. Weaker correlations were seen between the ACE-III and SSEVT (r = 0.161) **(B)**, and between the between the ACE-III and 4MT (r = 0.218) **(C)**. Scores on the Spatial Abilities and Practices Questionnaire (SAPQ) were most correlated with the 4MT (r = 0.199) **(D)**. See main text for further details of Pearson’s and Spearman’s correlation statistics. Solid blue line represents line of best fit. To avoid overplotting at shared, discrete positions, some data points were jittered, preserving vertical position while adjusting horizontal positions only marginally.

An overview of participant demographics and scores on each test and questionnaire for the healthy sample are shown in Table 1. The only sex difference observed was that males rated their spatial ability on the Spatial Abilities and Practices Questionnaire (SAPQ) higher than females did (t287 = 4.36, *p* = 2e-5, Cohen’s d = 0.516), which is in line with previous finding of sex differences in self-report of spatial abilities (Weiss et al., 2003) (Additional potential sex differences were further explored using regression analyses, but none emerged). The main focus of this report is the associations with age, education, and cognitive impairment of the three cognitive tests (4MT, SSEVT, and ACE-III). We note here at the outset that 4MT and SSEVT scores were more positively correlated with each other (*N* = 358, *r* = 0.334, *p* = 8e-11, rho = 0.330, *p* < 1e-10, Figure 4A) than with ACE-III (SSEVT: *N* = 344, *r* = 0.161, *p* = 0.003, rho = 0.158, *p* = 0.003; 4MT: *N* = 352, *r* = 0.218, *p* = 3.6e-5, rho = 0.231, *p* = 1.4e-5, Figures 4B, C). These results are consistent with the 4MT and our novel SSEVT being both designed to tap hippocampal-dependent cognition. We first consider associations of the cognitive tests with age.

**TABLE 1:** Descriptive statistics for each variable in the healthy sample.

	Males		Females		Total		Difference?
	*N*	Mean (SD)	*N*	Mean (SD)	*N*	Mean (SD)	
Age	161	43.78 (22.96)	215	41.12 (22.24)	376	42.26 (22.56)	t = l.13 *p* = 0.261
Education	161	2.85 (l.00)	215	2.72 (1.00)	376	2.77 (1.00)	t = l.24 *p* = 0.218
4MT	161	10.12 (2.82)	214	9.93 (2.72)	375	10.01 (2.76)	t = 0.67 *p* = 0.506
SSEVT	151	8.13 (2.22)	207	8.41 (2.38)	358	8.30 (2.31)	t = l.15 *p* = 0.252
ACE-III	150	93.04 (4.62)	202	93.30 (4.91)	352	93.19 (4.78)	t = 0.50 *p* = 0.616
SAPQ	124	55.69 (9.30)	165	50.73 (9.95)	289	52.86 (9.97)	t = 4.36 *p* = 2e-5**
SNSEQ	152	56.69 (8.93)	209	58.19 (8.92)	361	57.56 (8.94)	t = l.58 *p* = 0 116
ULS	133	24.73 (11.14)	182	22.76 (11.65)	315	23.59 (11.46)	t = l.52 *p* = 0.130
SWLS	133	24.47 (6.49)	180	25.73 (5.22)	313	25.20 (5.82)	t = l.83 *p* = 0.068

Overview of participant demographics (Age, Education) and scores on the cognitive tests [Four Mountains Test (4MT), Spaces and Sequences Episodic Video Task (SSEVT), Addenbrooke’s Cognitive Examination III (ACE-III)] and questionnaires [Spatial Abilities and Practices Questionnaire (SAPQ), Social Networks and Social Embeddedness Questionnaire (SNSEQ), UCLA Loneliness Scale (ULS), Satisfaction with Life Scale (SWLS)] for males and females separately and together. Where the value for a variable was missing for a participant, their score would be excluded from that variable alone, whilst retaining their other scores. A maximum of 87 subjects were excluded in this way (SAPQ, *N* = 289). Males scored higher on the SAPQ but no other sex differences were found. **p < 0.001.

### 3.2 Age-related decline in test performance was more pronounced in the 4MT and SSEVT than the ACE-III

We hypothesized (see section “1.6 Study rationale and aims: key points”) that episodic and spatial memory abilities, and thus 4MT and SSEVT scores, would appreciably decline with age. The healthy sample was divided into three groups: Young, 18–35; Middle-aged, 36–61; and Older people, 62–89 (Table 2).

**TABLE 2:** Descriptive statistics and scores on the 4MT, SSEVT, and ACE-III for the different age groups.

	Young	Middle-aged	Older	Difference?
*N*	187	74	115	–
Mean age (SD)	21.41 (2.84)	50.87 (8.37)	70.62 (6.61)	F = 3148.51, *p* = 2e-113**
Mean education (SD)	3.04 (0.47)	2.99 (1.09)	2.19 (1.29)	F = 23.02, *p* = 2e-9**
Mean 4MT score (SD)	11.15 (2.34)	9.38 (1.09)	8.55 (2.65)	F = 40.93, *p* = 3e-15**
Mean SSEVT score (SD)	9.09 (2.08)	8.14 (2.29)	6.93 (2.09)	F = 35.12, *p* = 2e-13**
Mean ACE- III score (SD)	93.90 (4.21)	92.87 (5.44)	92.01 (5.11)	F = 5.06, *p* = 0.007*

Young (18–35); Middle-aged (36–61); Older (62–89). *p < 0.05, **p < 0.001.

**FIGURE 5:**
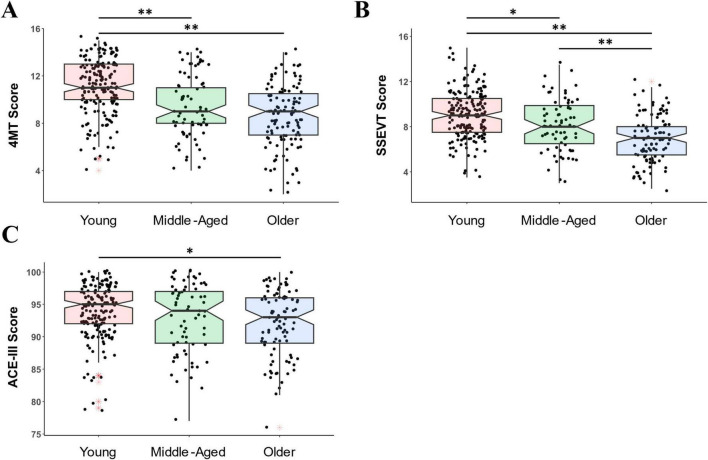
The 4MT and SSEVT were more sensitive to aging than the ACE-III. **(A)** On the 4MT, the young group scored higher than both Middle-aged and Older groups. The maximum score on the 4MT was 15. **(B)** On the SSEVT, the young group scored higher than both Middle-aged and Older groups, and the Middle-aged scored higher than the Older group. The maximum score on the SSEVT was 17. **(C)** On the ACE-III (which has a maximum score of 100), the only group difference was that the Young group scored somewhat higher than Older group. Shown are boxplots with boxes representing 25th percentile (bottom), 75th percentile (top) and median (central line and notches). Whiskers depict the minimum and maximum calculated values for each group (minimum: Q 1 -l.5*IQR; maximum Q3 +l.5*IQR). Black dots represent individual datapoints. Red asterisks signpost outliers. Young = 18–35 years; Middle-Aged = 36–61 years; Older = 62–89 years. *p < 0.05, **p < 0.001.

There was a clear effect of age group on 4MT scores [F(2, 174) = 40.93, *p* = 3e-15, η2 = 0.181, Figure 5A]. Young people scored higher than both Middle-aged (t = 4.94, *p* = 8e-6, Cohen’s d = 0.724), and Older groups (t = 8.76, p = 7e-16, Cohen’s d = 1.057), with no difference between the Middle-aged and Older people (*p* = 0.085).

There was also a clear effect of age group on SSEVT scores [F(2, 164) = 35.11, *p* = 2e-13, η2 = 0.161, Figure 5B), with Older people scoring lower than both Middle-aged (t = 3.52, *p* = 0.002, Cohen’s d = 0.557) and Young participants (t = 8.37, *p* = 6e-14, Cohen’s d = 1.036), and Middle-Aged people also scoring lower than Young participants (t = 3.02, *p* = 0.009, Cohen’s d = 0.443).

Compared to 4MT and SSEVT, the effect of age group on ACE-III scores (Figure 5C) was much weaker [F(2, 150) = 5.063, *p* = 0.007, η2 = 0.029], with the only group difference being between Young and Older people, (t = 3.09, *p* = 0.007, Cohen’s d = 0.417, other comparison *p*-values > 0.328.

Consistent with these group differences, the negative correlation between age and performance was higher for 4MT (*N* = 375, *r* = −0.451, *p* = 3e-20, rho = −0.425, *p* = 7e-18) and SSEVT (*N* = 358, *r* = −0.418, *p* = 1e-16, rho = −0.404, *p* = 2e-15) than for ACE-III (*N* = 352, r = −0.185, *p* = 5e-4, rho = −0.122, *p* = 0.022). In summary, these findings are consistent with ACE-III being a test of diverse cognitions, including those (e.g., semantic memory) relatively well-preserved in aging, and with SSEVT and 4MT being tests of more specific, hippocampal-dependent cognitions, and thus expected to decline more severely with aging.

In exploratory analyses, we also examined relationships between the five individual sections of the SSEVT and other measures. A negative correlation with age was clearly seen for Section 1–4 scores (*N* = 357, Section 1: r = −0.283, *p* = 5e-8, rho = −*0.279*, *p* = 8e-8; Section 2: r = −0.220, *p* = 3e-5, rho = −*0.219*, *p* = 3e-5; Section 3: r = −0.218, *p* = 3e-5, rho = −*0.222*, *p* = 2e-5; Section 4: r = −0.286, *p* = 4e-8, rho = −*0.255*, *p* = 1e-6), but this was not reliable for section 5 (r = −0.089, *p* = 0.094, rho = −*0.071*, *p* = 0.181). Broadly similarly, positive correlations with 4MT were seen for Sections 1–4 (*N* = *357*, all *p* < 0.005), but this was not reliable for section 5 (r = 0.088, *p* = 0.098, rho = *0.075*, *p* = 0.159) (Table 3).

**TABLE 3:** Correlations between Spaces and Sequences Episodic Video Task (SSEVT) Section scores and other variables.

Variable	Test	4MT	SAPQ	S1	S2	S3	S4	S5
4MT	Pearson’s	–	–	–	–	–	–	–
	Spearman’s	–	–	–	–	–	–	–
SAPQ	Pearson’s	r = 0.199 ***p* = 7e-4**	–	–	–	–	–	–
	Spearman’s	rho = *0.207* ***p* = 4e-4**	–	–	–	–	–	–
Sl	Pearson’s	*r = 0.155* ***p* = 0.003**	r = 0.041 *p* = 0.489	–	–	–	–	–
	Spearman’s	rho = 0.149 ***p* = 0.005**	rho = 0.048 *p* = 0.424	–	–	–	–	–
S2	Pearson’s	r = 0.162 ***p* = 0.002**	r = 0.157 ***p* = 0.008**	r = 0.113 ***p* = 0.033**	–	–	–	–
	Spearman’s	rho = 0.157 ***p* = 0.003**	rho = 0.144 ***p* = 0.0l5**	rho = 0.090 *p* = 0.090	–	–	–	–
S3	Pearson’s	r = 0.167 ***p* = 0.001**	r = 0.065 *p* = 0.277	*r* = 0.152 ***p* = 0.004**	r = -5e-20 *p* = 1.000	–	–	–
	Spearman’s	rho = 0 158 ***p* = 0.003**	rho = 0.080 *p* = 0.180	rho = 0.156 ***p* = 0.003**	rho = -0.010 *p* = 0.853	–	–	–
S4	Pearson’s	r = 0.297 ***p* = le-10**	r = 0.177 *p* = 0.003	r = 0.207 ***p* = 8e-5**	r = 0.092 *p* = 0.083	r = 0.191 ***p* = 3e-4**	–	–
	Spearman’s	rho = 0.305 ***p* = 0.4e-9**	rho = 0.183 ***p* = 0.002**	rho = 0.195 ***p* = 2e-4**	rho = 0.082 *p* = 0.120	rho = 0.184 ***p* = 5e-4**	–	–
SS	Pearson’s	r = 0.088 *p* = 0.098	r = 0.038 *p* = 0.528	r = 0.089 *p* = 0.094	r = –0.008 *p* = 0.883	r = 0.060 *p* = 0.259	r = 0.090 *p* = 0.089	–
	Spearman’s	rho = 0.075 *p* = 0.159	rho = 0.057 *p* = 0.336	rho = 0.097 *p* = 0.067	rho = –0.030 *p* = 0.575	rho = 0.068 *p* = 0.199	rho = 0.093 *p* = 0.081	–

Table 3 shows the relationships between the five sections of the Spaces and Sequences Episodic Video Task (SSEVT) (Sl–5) and with the Four Mountains Test (4MT) and Spatial Abilities and Practices Questionnaire (SAPQ). *p* < 0.05 are in bold.

### 3.3 Education positively predicted performance on ACE-III and 4MT, but not SSEVT

We next explored potential education effects. Education levels differed between the three age groups, F(2,136) = 23.02, *p* = 2e-9, η*^2^* = 0.149, with older people having less formal education than both middle-aged (t = 4.55, *p* = 3e-5) and younger (t = 6.80, *p* = 1e-9) people, reflecting generational changes in United Kingdom. Accordingly, straightforward between-group analysis across educational levels (Table 4) is potentially misleading. To try tease out independent associations of age and education, and check if the age-related associations above (see section “3.2 Age-related decline in test performance was more pronounced in the 4MT and SSEVT than the ACE-III”) were robust, we ran multiple regressions on the entire participant sample, with age and education as co-predictors of cognitive test performance.

**TABLE 4:** Age and cognitive test scores on the Four Mountains Test (4MT), Spaces and Sequences Episodic Video Task (SSEVT), and Addenbrooke’s Cognitive Examination-III (ACE-III) across education levels.

Education level	Age		4MT		SSEVT		ACE-III	
	*N*	Mean (SD)	*N*	Mean score (SD)	*N*	Mean score (SD)	*N*	Mean score (SD)
0	17	71.24 (7.73)	17	7.06 (2.30)	11	6.55 (1.62)	13	91.46 (5.03)
1	34	60.88 (18.58)	34	8.35 (3.07)	31	7.42 (2.38)	25	89.24 (5.04)
2	36	54.58 (21.54)	36	9.81 (2.71)	30	7.72 (1.94)	31	91.71 (5.87)
3	220	33.35 (20.02)	219	10.56 (2.50)	217	8.69 (2.32)	216	93.60 (4.32)
4	69	47.90 (18.50)	69	9.90 (2.78)	69	7.97 (2.22)	67	94.36 (4.68)
Difference?	F = 71.03, *p* = l e-25**		*F* = 11.82, *p* = 2e-7**		F = 6.53, *p* = 2e-4**		F = 5.95, *p* = 5e-4**	
Group differences	0 > 2**; 0 > 3**; 0 > 4**; 1 > 3**; 1 > 4*; 2 > 3**; 4 > 3**		2 > 0*; 3 > 0**; 4 > 0*; 3 > 1*		3 > 0*		3 > 1*; 4 > 1**	

*p < 0.05, **p < 0.001 (Games-Howell *post hoc* group differences). Simple presentation of variation in scores on the cognitive tests according to level of Education (more education associated with higher scores). Importantly however, complicating interpretation, reflecting generational changes in UK, older people had less formal education than both middle-aged and younger people. See main text for further discussion and analysis of associations of age and education with cognitive test scores. Education Level Key: 0 (No formal educational qualifications); 1 (GCSE’s or equivalent); 2 (A-Levels or equivalent); 3 (Undergraduate degree or equivalent); 4 (Postgraduate degree or equivalent).

In a significantly predictive model of ACE-III [*F*(2,349) = 14.33, AdjR^2^ = 0.071, *p* = 1e-6], education was more positively predictive (β = 0.212, *p* < 0.00009), than age was negatively predictive (β = -0.126, *p* = 0.02), of ACE-III scores.

This pattern was different to that for 4MT [*F*(2,372) = 51.54, AdjR^2^ = 0.211, *p* = 2e-20], where age was strongly negatively predictive (β = −0.412, *p* = 6e-19), and education weakly positively predictive (β = 0.118, *p* = 0.016), of 4MT scores.

As regards our novel SSEVT [*F*(2,355) = 37.71, AdjR^2^ = 0.171, *p* = 1e-15], age strongly negatively predicted (β = −0.412, *p* = 5e-15), while education did *not* predict (β = 0.02, *p* = 0.67), SSEVT scores.

We note two points in summary. The first point is merely confirmatory of our age-related analyses above (see section “3.2 Age-related decline in test performance was more pronounced in the 4MT and SSEVT than the ACE-III,” Figure 5); that similarly large age-related reductions in performance were seen for 4MT and SSEVT (both β*s* = −0.41), while a much smaller age-related effect was seen for ACE-III (β = −0.126). The second point is that the potential influence of education upon task performance varied across the three tasks. This influence was clearest for ACE-III (β = 0.212), intermediate for 4MT (β = 0.118), while there was no sign that education boosted SSEVT performance (β = 0.02, *p* = 0.67).

The lack of association of level of education with SSEVT scores reported here is consistent with our design aim that this task be minimally dependent upon education. However, we acknowledge sample limitations, and the need to replicate this result with a larger sample with a good variance in education levels across all ages.

### 3.4 Social embeddedness, loneliness, and life satisfaction cannot predict cognitive performance

In our sample, older people were not less socially embedded (*N* = 361, SNSEQ, r = 0.003, *p* = 0.950, rho = −0.019, *p* = 0.716) or more lonely (*N* = 315, ULS, r = −0.036, *p* = 0.524, rho = −0.035, *p* = 0.532), but were more satisfied with life (*N* = 313, r = 0.128, *p* = 0.023, rho = 0.118, *p* = 0.037). Social embeddedness (SNSEQ score) was not correlated with 4MT (*N* = 360, r = −0.016, *p* = 0.768, rho = 0.004, *p* = 0.938) or SSEVT performance (*N* = 357, r = −0.022, *p* = 0.677, rho = −0.014, *p* = 0.792), but was positively correlated with SAPQ scores (*N* = 289, r = 0.280, *p* = 1e-6, rho = 0.291, *p* = 5e-7) and (weakly) with ACE-III scores (*N* = 347, r = 0.106, *p* = 0.049, rho = 0.123, *p* = 0.022). Neither the SWLS nor ULS were predictive of any measure of cognitive performance.

### 3.5 Spatial Abilities and Practices Questionnaire (SAPQ)

There were no differences between age-groups on overall SAPQ score, [*F*(2,137) = 0.961, *p* = 0.385, η*^2^* = 0.006]. Age did not predict SAPQ scores in the whole sample (β = −0.083, *p* = 0.160), in men (β = 0.161, *p* = 0.075) or in women (β = 0.033, *p* = 0.671). However, when “change” scores (i.e., how much each rated ability had changed in the past year) were examined in isolation from overall SAPQ score, age was negatively correlated with reported change score (*N* = 242, r = −0.365, *p* = 4.9e-9, rho = −0.410, *p* = 3.1e-11). That is, younger people tended to report more improvements, and older people more declines, over time. The SAPQ was positively correlated with performance on the 4MT (*N* = 288, r = 0.199, *p* = 7e-4), (Figure 4D), and SSEVT (*N* = 285, *r* = 0.178, *p* = 0.002), but not ACE-III (*N* = 276, r = 0.114, *p* = 0.059). Exploratory analysis found positive correlations of SAPQ scores with SSEVT scores on Section 2 spatial context, (*N* = 285, r = 0.157, *p* = 0.008, rho = 0.144, *p* = 0.015) and Section 4 event order, (*N* = 285, r = 0.177, *p* = 0.003, rho = 0.183, *p* = 0.002) of the SSEVT, but these were weak on the other Sections (Sections 1, 2, and 5, all *p* > 0.277). Three separate multiple regressions were run with each test score, and age, as co-predictors of overall SAPQ score. Consistent with the opening results of this section, age never significantly predicted SAPQ score. Only 4MT scores (β = 0.206, *p* = 0.002), and SSEVT scores (β = 0.174, *p* = 0.009), but not ACE-III score (β = 0.103, *p* = 0.093, overall model not significant), could significantly predict overall SAPQ performance.

### 3.6 The 4MT and SSEVT (but not ACE-III) discriminated MCI patients from healthy aging controls

As proof of concept of the ability of 4MT and SSEVT to discriminate healthy from pathological aging (section “1.6 Study rationale and aims: key points” above), we compared a small group of MCI patients (*n* = 10) to a sample of healthy aging controls (*n* = 78), matched for age and education. For further details on the matching and group scores, please see Table 5. As intended, the MCI group did not differ from controls in age (t_86_ = 1.04, *p* = 0.321), sex (X*^2^* = 0.023, *p* = 0.879), or education (t_86_ = 1.45, *p* = 0.175).

**TABLE 5:** Participant demographics and scores in the MCI (*N* = 10) and matched healthy control group (max *N* = 78) on the Four Mountains Test (4MT), Spaces and Sequences Episodic Video Task (SSEVT), Addenbrooke’s Cognitive Examination III (ACE-III), Spatial Abilities and Practices Questionnaire (SAPQ), and Social Networks and Social Embeddedness Questionnaire (SNSEQ).

	MCI patients				
	*N*	Mean age (SD)	Sex (% female)	Mean education (SD)	Mean score (SD)
4MT	10	73.30 (8.47)	50	1.60 (1.51)	6.20 (2.74)
SSEVT	7	77.57 (5.91)	57. 14	1.71 (1.25)	4.7 1 (0.49)
SSEVT (specific matched)	7	77.57 (5.91)	57.14	1.71 (1.25)	4.71 (0.49)
ACE-III	10	73.30 (8.47)	50	1.60 (1.51)	88.00 (11.99)
SAPQ	10	73.30 (8.47)	50	1.60 (1.51)	45.40 (16.97)
SNSEQ	10	73.30 (8.47)	50	1.60 (1.51)	51.00 (8.10)
**Healthy controls**					**Difference?**
***N***	**Mean age (SD)**	**Sex (% female)**	**Mean education (SD)**	**Mean score (SD)**	
78	70.41 (6.18)	47.44	2.32 (1.21)	8.54 (2.56)	t = 2.56 *p* = 0.026*
69	70.58 (5.91)	49.28	2.46 (1.16)	6.90 (2.16)	t = 6.86 *p* = 3e-8**
38	74.74 (4.65)	55.26	2.03 (1.17)	6.43 (2.02)	t = 4.58 *p* = 5e-5**
60	71.23 (8.47)	46.67	2.57 (1.14)	91.80 (5.31)	*t* = *0.99 p* = 0.348
60	71.10 (6.39)	46.67	2.52 (1.11)	51.87 (9.09)	t = l.18 *p* = 0.267
70	70.84 (6.27)	48.57	2.47 (l.15)	57.99 (9.96)	t = 2.47 *p* = 0.028*

*p < 0.05, **p < 0.001, Welch *t*-test. The healthy control group was created by matching healthy controls to individual mild cognitive impairment (MCI) patients based on age (+3 years) and education (grouped into 0, 1–2, and 3–4). This gave an overall control group of 78 participants. A separate group of controls (*N* = 38) was also created specifically matched to the seven MCI patients who completed the Spaces and Sequences Episodic Video Task (SSEVT), due to the smaller sample size of this group.

**FIGURE 6:**
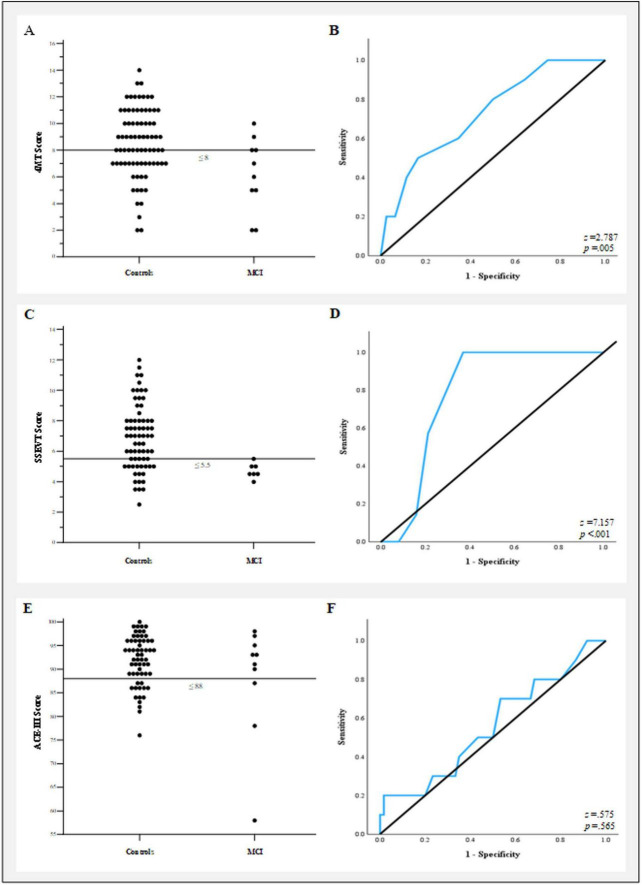
In initial, proof-of-concept exploratory comparison, MCI patients perform significantly worse on the 4MT and SSEVT, but not on the ACE-III. Threshold plot showing 4MT scores for control group (left) and MCI patient group (right), and recommended cut-off associated with best between-group differentiation (score ≤ 8, thin horizontal black line, 80% sensitivity and 50% specificity.) **(B)** ROC plot for 4MT (AUC: 0.729, *p* = 0.005). **(C)** As for **(A)**, threshold plot showing SSEVT scores for both groups, and recommended cut-off associated with best between-group differentiation (score ≤ 5.5, 100% sensitivity and 63% specificity). **(D)** ROC plot for SSEVT (AUC: 0.835, *p* < 0.001). **(E)** Threshold plot for ACE-III scores, for score ≤ 88 [a recommended cutoff for MCI detection in Beishon et al. (2019)], associated with 30% sensitivity and 69% specificity. **(F)** ROC plot for ACE-III, which did not deviate from chance classification (AUC: 0.560, *p* = 0.565).

As expected, 4MT scores were lower in MCI patients than healthy aging controls (t_86_ = 2.56, *p* = 0.026, Cohen’s d = 0.882, Figure 6A). Similarly, as expected, SSEVT scores were lower in MCI patients than these controls (t_74_ = 6.86, *p* = 3e-8, Cohen’s d = 1.397, Figure 6B). Exploratory section-by-section analysis is shown in Table 6. The sections with the largest relative impairments in MCI were: Section 1 (character-associations, Cohen’s d = 1.216); section 5 (room order, Cohen’s d = 0.694), and Section 4 (event order, Cohen’s d = 0.511), but only that for Section 1 was statistically significant (1: t_74_ = 3.77, *p* = 0.004; 5: t_74_ = 1.98, *p* = 0.082; 4: t_74_ = 1.33, *p* = 0.223). An appreciably larger sample is needed for reliable section-by-section analysis. Given the limited MCI sample available for the SSEVT (*N* = 7), we compared these results with a more closely-matched control sample (*N* = 38: MCI vs HA: age (t_43_ = 1.20, *p* = 0.266), sex (X^2^ = 0.09, p = 0.933), education (t_43_ = 0.61, *p* = 0.558), which produced a similar result (t_39_ = 4.58, *p* = 5e-5, Cohen’s d = 1.172). ACE-III scores did not significantly differ between MCI patients and controls (t_10_ = 0.99, *p* = 0.348, Cohen’s d = 0.410, Figure 6C). This may partly reflect a lack of statistical power in this small sample.

**TABLE 6:** Participant scores in the mild cognitive impairment (MCI) (*N* = 7) and healthy control group [*N* = *69* for Spaces and Sequences Episodic Video Task (SSEVT)] on the sections of the SSEVT.

Section	Mean score (SD)		Difference?
	MCI	Healthy controls	
1	0.57 (0.54)	1.42 (0.83)	t = 3.77 *p* = 0.004*
2	1.29 (0.49)	1.58 (0.88)	t = 1.38 *p* = 0.196
3	0.86 (0.39)	1.12 (0.98)	t = 1.40 *p* = 0.181
4	0.71 (0.76)	1.12 (0.81)	t = 1.33 *p* = 0.223
5	1.29 (0.49)	1.68 (0.64)	t = 1.98 *p* = 0.082

*p < 0.05. The healthy control group was created by matching healthy controls to the ten individual mild cognitive impairment (MCI) patients based on age (+3 years) and education (grouped into 0, 1–2, and 3–4). This gave a total of *N* = 78, with 69 of these controls having completed the Spaces and Sequences Episodic Video Task (SSEVT).

The Four Mountains Task performance differentiated the MCI patients and controls with an AUC (ROC, DeLong et al., 1988) of 0.729 (*z* = 2.787, *p* = 0.005); a 4MT score of ≤ 8 was associated with 80% sensitivity and 50% specificity (Figures 6A, B). For the SSEVT, the AUC was 0.835, (*z* = 7.157, *p* < 0.001); SSEVT scores of ≤ 5.5 were associated with 100% sensitivity and 63% specificity (Figures 6C, D). In contrast, ACE-III test performance did not achieve better-than-chance discrimination (AUC = 0.560, *z* = 0.575, *p* = 0.565) (Figures 6E, F).

In summary, our findings provide some preliminary, proof-of-concept evidence that 4MT and SSEVT are useful in discriminating pathological from healthy cognitive aging. Further, since most MCI patients typically progress to a diagnosis of AD, these tests may be useful in assessing early stages of AD.

### 3.7 MCI patients vs. healthy aging controls: questionnaire results

Mild cognitive impairment patient questionnaire data were available for the measures of social embedding (SSNEQ), and spatial abilities and practices (SAPQ). MCI patients were less socially embedded than their healthy aging controls (t_78_ = 2.47, *p* = 0.028). This is potentially consistent with social isolation as a risk factor for dementia (see section “2.2 Tests and questionnaires” above). However, caution is clearly merited: (1) the sample size of the patients is small (*n* = 10); (2) in this cross-sectional study, the lower social embedding at the MCI diagnosis stage may reflect a compensatory response to cognitive decline, rather than a risk factor. Neither overall SAPQ scores (t_68_ = 1.18, *p* = 0.267) nor SAPQ change scores (t_35_ = 0.43, *p* = 0.673) differed between MCI patients and controls.

## 4 Discussion

### 4.1 The SSEVT and 4MT were sensitive to aging

In the same participants, age-related decline in performance was greater on the tasks tapping spatial and episodic memory (4MT: allocentric spatial memory; SSEVT, spatial and episodic/sequence memory) than on the ACE-III. This is consistent with: (a) ACE-III being a composite test of diverse cognitive functions, including linguistic ability and semantic memory, which are relatively well-preserved in aging; (b) SSEVT and 4MT tapping hippocampal-dependent cognition, which would be expected, given hippocampal age-related neurodegeneration, to decline more severely with age.

Accordingly, SSEVT and 4MT may be useful tasks in assessments of cognitive aging. These could include correlative and intervention-type studies examining the potential “anti-aging” effects of factors such as exercise, stress reduction, good sleep, and good diet which are expected to impact upon hippocampal tissue and hippocampal-dependent cognition. One advantage of both these tasks is that they both use forced-choice question formats, and straightforward scoring, thus potentially enabling automatic and/or remote testing. In so much as both the 4MT and SSEVT test spatial memory, the marked age-related diminution in performance on these tasks is consistent with the age-associated decline in use of spatial strategies reported in a very large scale study of a mobile app-based video game (“Sea Hero Quest”) (West et al., 2023).

In contrast to these objective tests tapping spatial memory ability (4MT: allocentric spatial memory; SSEVT: spatial and episodic/sequence memory), our novel questionnaire assessing “everyday” spatial abilities (SAPQ) was not very sensitive to age. Change over the last year, (the young tending to improve, the old tending to worsen), rather than absolute score, was correlated with age.

### 4.2 Education positively predicted performance on ACE-III and 4MT, but not SSEVT

We found that education levels predicted performance on ACE-III and 4MT in a positive manner, (the higher the education level, the higher the scores), but did not predict SSEVT scores. The finding that education level did not predict SSEVT scores aligns with our intention for SSEVT to be minimally dependent upon education-based expertise. We intended that the SSEVT, including its instructions and questions, should be simple to understand, and that success on the SSEVT should be resistant to education-reliant strategies. This non-association with education is thus encouraging, but we should acknowledge a caveat. In our sample, older people tended to be less educated than our young and middle-aged groups. It will be important to replicate the non-association of education with SSEVT scores in a larger sample with a good variance in education levels across all ages.

A task that is relatively easy to understand, and where education cannot confer any additional benefits to task performance (e.g., in verbalizing events) would be advantageous, since the need to take account of education is a complicating factor in tests of cognitive aging and AD diagnostics. To give just one example relevant here, one study used five screening tests for diagnosing AD, and found that with all of them, but especially the ACE-III, diagnostic accuracy for AD was higher in patients with full primary education than in the less educated group (Matias-Guiu et al., 2017).

### 4.3 The 4MT and SSEVT discriminated MCI from healthy aging

The 4MT and SSEVT were also designed with a view to detecting early stages of pathological aging, such as in Alzheimer’s disease. Testing spatial and/or sequence memory has also been used by other groups with a view to detecting (early) Alzheimer’s disease (e.g., Bellassen et al., 2012; Tu et al., 2017; Coughlan et al., 2019; Howett et al., 2019; Park and Lee, 2021; Schöberl et al., 2020; Coughlan et al., 2023).

As an initial proof-of-concept test of this idea, we examined MCI-vs.-HA discrimination in three cognitive tests: the 4MT, SSEVT, and ACE-III. Consistent with previous evidence for such detection (e.g., Moodley et al., 2015; Wood et al., 2016), the 4MT did discriminate the MCI and HA groups. We also found that our novel task, the SSEVT, did discriminate the MCI and HA groups. In contrast, the ACE-III did not significantly discriminate the MCI and HA groups. ACE-III was likely not designed to detect specifically early-AD pathology (rhinal cortices, hippocampus), and measures some cognitive domains (attention, verbal fluency, language) generally impacted only later in AD disease progression (Grady et al., 1988; Irwin et al., 2018), and may not accurately capture MCI-specific impairments (Beishon et al., 2019). While our results are preliminary, they provide some initial proof-of-concept encouragement for the possibility of using the 4MT (further to that summarized in see section “1.5 Could tests tapping the hippocampus fill the gap?”) and SSEVT as screening tools for pathological aging including that due to early AD.

### 4.4 Social embedding

In this sample, MCI patients were less socially embedded (SNSEQ) than their age-matched controls. Our small MCI sample size, cross-sectional design, and opportunistic sampling prohibits any conclusive inferences. We simply note here that this result is potentially consistent with previous research suggesting that social isolation is a risk factor for cognitive deterioration [sections 2.2.5/–6 see also (e.g., Pereda-Pérez et al., 2013; Wang et al., 2019; Drinkwater et al., 2022)], but also that social isolation can be a consequence of impaired cognition, (e.g., Hultsch et al., 1999). In the healthy sample, the degree of social embedding was not correlated with 4MT or SSEVT performance, and only weakly positively correlated with ACE-III performance (r = 0.106, rho = 0.123). This issue should be investigated in larger, older-biased samples, with recruitment including more socially-isolated individuals than was possible here.

### 3.4 Limitations and future directions

While middle-aged people were under-represented in our sample, the sample size in the first part of this study is likely sufficient for some confidence in our findings of much greater age-related decline in performance on the 4MT and SSEVT than on the ACE-III. Effect sizes were fairly large for 4MT and SSEVT, and much smaller for ACE-III. However, as we have acknowledged above, the differing patterns of association between task performance and education, namely small effect sizes for ACE-III and 4MT, and no effect for SSEVT, warrant further study in a sample with greater variance in education at all ages.

As we outlined in see section “1.4 Some limitations of existing screening tools for AD and cognitive aging” above, our aims in exploring 4MT and SSEVT were to overcome some of the limitations associated with verbal episodic memory tasks, which lack ecological validity, can be complex to score, and strongly modulated by factors such as sex and verbal intelligence. Nevertheless, a test of verbal episodic memory could have provided a useful additional comparator task in the present study by which to evaluate the success of 4MT and SSEVT. We aim to address this in future work.

The rather small patient sample, defined without necessary accompanying biomarkers according to the Albert et al. (2011) criteria, in this study’s second part, is the present study’s main limitation, limiting our findings to being initial proof-of-concept data. Nevertheless, it seems encouraging that the SSEVT may have value for future examination of both modulators of healthy aging, and the discrimination of healthy from pathological cognitive aging. We acknowledge the limitation that our healthy aging sample was largely White British, and our MCI sample entirely White British. It would be good to replicate the results obtained here with different ethnocultural samples.

This is the first report on the use of the SSEVT. We envisage potential improvements and adaptations can be made to its use. Although the SSEVT does not directly require facial recognition, participants will benefit from the ability to recognize faces. Color blindness could also potentially impact performance negatively. This could be addressed in future work by the addition of pre-test screens for prosopagnosia (face recognition) and color blindness. To suit particular study purposes, the number of presentations can be increased, and the length of the delay between presentation and test can be lengthened. In ongoing work examining consolidation for instance, we have used delays of 30 min and 2 weeks after three presentations of the SSEVT video, with separate sets of questions for the 30 min and the 2 weeks delay.

### 4.6 Conclusion

The Four Mountains Test and SSEVT are highly sensitive to a diminution in cognition associated with advancing age, and detectable at middle age. Since 4MT and SSEVT were designed to test hippocampal-dependent cognition, we interpret this age-related diminution as attributable (at least partly) to hippocampal atrophy being prominent in aging and present at middle age. ACE-III, a composite test of diverse cognitive functions, was much less age-modulated than 4MT and SSEVT. The degree to which education appeared to boost test performance was variable across the tasks: moderate for ACE-III, weak for 4MT, and minimal/absent for SSEVT. The seemingly minimal modulation of SSEVT scores by education and sex may confer extra value for its potential use in testing factors promoting healthy aging, and in discriminating healthy from pathological cognitive aging.

## Data Availability

The raw data supporting the conclusions of this article will be made available by the authors, without undue reservation.
