# Supplementary material for: Exploring the sensitivity of episodic and spatial memory tests to healthy and pathological cognitive aging
Source: Front Aging Neurosci. 2025 Jun 20;17:1547834. doi: 10.3389/fnagi.2025.1547834 (PMC12226565; doi:10.3389/fnagi.2025.1547834)
Supplement: Supplementary file 1 [file Data_Sheet_1.pdf]

Spatial Abilities and Practices Questionnaire

This questionnaire asks about your spatial abilities and practices in everyday life. There is no time limit so please feel free to take your time. We would advise that, if possible, you fill out this questionnaire together with someone who knows you well.

Have you filled out this questionnaire with someone who knows you well?

YES NO

Read each statement carefully. Answer to what extent you agree with each statement by choosing the answer that most applies to you.

After each statement, we also ask if you think that there has been a change in your spatial ability in the last 12 months.

1) I am good at recognising a place (eg. town square, building) even when I approach it from a new direction.

STRONGLY AGREE AGREE UNDECIDED DISAGREE STRONGLY DISAGREE

Has your spatial ability in this respect changed over the last 12 months? Has it got better, is it the same as before, or is it worse?

BETTER SAME WORSE

2) When leaving or returning home, I generally have a good idea of the direction between my home and destination.

STRONGLY AGREE AGREE UNDECIDED DISAGREE STRONGLY DISAGREE

Has your spatial ability in this respect changed over the last 12 months? Has it got better, is it the same as before, or is it worse?

BETTER SAME WORSE

3) When I walk out of a large shop or shopping centre, I sometimes find I am taking the wrong direction from the one I intended.

STRONGLY AGREE AGREE UNDECIDED DISAGREE STRONGLY DISAGREE

Has your spatial ability in this respect changed over the last 12 months? Has it got better, is it the same as before, or is it worse?

BETTER SAME WORSE

4) I have difficulty accurately visualizing in my mind's eye the local walking routes (e.g. to shops, pubs, parks, restaurants) to and from my home.

STRONGLY AGREE AGREE UNDECIDED DISAGREE STRONGLY DISAGREE

Has your spatial ability in this respect changed over the last 12 months? Has it got better, is it the same as before, or is it worse?

BETTER SAME WORSE

5) I find it easy to visualize in my mind's eye the routes to places further afield (e.g. to nearby towns).

STRONGLY AGREE AGREE UNDECIDED DISAGREE STRONGLY DISAGREE

Has your spatial ability in this respect changed over the last 12 months? Has it got better, is it the same as before, or is it worse?

BETTER SAME WORSE

6) When a walking route I usually take is completely blocked off (e.g. for maintenance, treefalls), I find it difficult to work out a new route.

STRONGLY AGREE AGREE UNDECIDED DISAGREE STRONGLY DISAGREE

Has your spatial ability in this respect changed over the last 12 months? Has it got better, is it the same as before, or is it worse?

BETTER SAME WORSE

7) I find it easy to remember precisely where the car is parked.

STRONGLY AGREE AGREE UNDECIDED DISAGREE STRONGLY DISAGREE

Has your spatial ability in this respect changed over the last 12 months? Has it got better, is it the same as before, or is it worse?

BETTER SAME WORSE

8) I stick to major walking routes, and avoid minor paths and shortcuts, to avoid getting lost.

STRONGLY AGREE AGREE UNDECIDED DISAGREE STRONGLY DISAGREE

Has your spatial ability in this respect changed over the last 12 months? Has it got better, is it the same as before, or is it worse?

BETTER SAME WORSE

9) When visiting new places, I prefer to be with people I know and follow them, rather than find my way myself as I feel that if I am alone, I may become lost.

STRONGLY AGREE AGREE UNDECIDED DISAGREE STRONGLY DISAGREE

Has your spatial ability in this respect changed over the last 12 months? Has it got better, is it the same as before, or is it worse?

BETTER SAME WORSE

10) It is difficult for me to find my bearings in a new town/city.

STRONGLY AGREE AGREE UNDECIDED DISAGREE STRONGLY DISAGREE

Has your spatial ability in this respect changed over the last 12 months? Has it got better, is it the same as before, or is it worse?

BETTER SAME WORSE

11) As a passenger in a car, I usually have to take the same route many times to remember it.

STRONGLY AGREE AGREE UNDECIDED DISAGREE STRONGLY DISAGREE

Has your spatial ability in this respect changed over the last 12 months? Has it got better, is it the same as before, or is it worse?

BETTER SAME WORSE

12) Before I go somewhere, I tend to visualise the different points along the journey where I need to make a decision (e.g. where I will need to go straight, left or right).

STRONGLY AGREE AGREE UNDECIDED DISAGREE STRONGLY DISAGREE

Has your spatial ability in this respect changed over the last 12 months? Has it got better, is it the same as before, or is it worse?

BETTER SAME WORSE

13) I can tell quite quickly that I am approaching a place I have been to before, even if I have only been there once or twice.

STRONGLY AGREE AGREE UNDECIDED DISAGREE STRONGLY DISAGREE

Has your spatial ability in this respect changed over the last 12 months? Has it got better, is it the same as before, or is it worse?

BETTER SAME WORSE

14) On an outing or holiday, having walked around visiting several locations (e.g. coffee shop, museum) I can generally calculate a shortcut route back to my starting point without consulting signs and maps.

STRONGLY AGREE AGREE UNDECIDED DISAGREE STRONGLY DISAGREE

Has your spatial ability in this respect changed over the last 12 months? Has it got better, is it the same as before, or is it worse?

BETTER SAME WORSE

15) I can find my way around places when travelling in the dark.

STRONGLY AGREE AGREE UNDECIDED DISAGREE STRONGLY DISAGREE

Has your spatial ability in this respect changed over the last 12 months? Has it got better, is it the same as before, or is it worse?

BETTER SAME WORSE

Supplementary Materials 2

Social Networks and Social Embeddedness Questionnaire

This questionnaire asks questions about your social circle. There is no time limit so feel free to take your time. We would advise that, if possible, you fill out this questionnaire together with someone who knows you well. Have you filled out this questionnaire with someone who knows you well?

YES   **NO**

Read each statement carefully. Answer to what extent you agree with each statement by choosing the answer that most applies to you.

After each statement, we also ask if you think that there has been a change in your social situation in the last 12 months.

	Strongly Disagree	*Disagree*	*Neither agree nor disagree*	*Agree*	*Strongly Agree*
**1. I would describe myself as very sociable.**	☐	☐	☐	☐	☐
**This statement has become less applicable to me in the last 12 months.**	☐	☐	☐	☐	☐
**2. I find it easy to meet new people.**	☐	☐	☐	☐	☐
**This statement has become less applicable to me in the last 12 months.**	☐	☐	☐	☐	☐
**3. There are people outside of my family that I can talk to about personal matters.**	☐	☐	☐	☐	☐
**This statement has become less applicable to me in the last 12 months.**	☐	☐	☐	☐	☐
**4. On an average week, I will only interact with my spouse and/or children.**	☐	☐	☐	☐	☐
**This has become more applicable to me in the last 12 months.**	☐	☐	☐	☐	☐
**5. I have friends outside of family members that I see on at least a fortnightly basis.**	☐	☐	☐	☐	☐
**This statement has become less applicable to me in the last 12 months.**	☐	☐	☐	☐	☐
**6. I can sometimes feel lonely.**	☐	☐	☐	☐	☐
**This statement has become more applicable to me in the last 12 months.**	☐	☐	☐	☐	☐
**7. I have less desire to meet new people than I used to.**	☐	☐	☐	☐	☐
**This statement has become even more applicable to me in the last 12 months.**	☐	☐	☐	☐	☐
**8. If I were to have a party, I can think of more than 5 people aside from family members that would attend.**	☐	☐	☐	☐	☐
**This statement has become less applicable to me in the last 12 months.**	☐	☐	☐	☐	☐

9. I tend to avoid social situations unless they are with my immediate family.	☐	☐	☐	☐	☐
This statement has become more applicable to me in the last 12 months.	☐	☐	☐	☐	☐
10. I'd rather stay at home than have day trips out.	☐	☐	☐	☐	☐
This statement has become more applicable to me in the last 12 months.	☐	☐	☐	☐	☐
11. I've fallen out of touch with many of my closest friends.	☐	☐	☐	☐	☐
This statement has become more applicable to me in the last 12 months.	☐	☐	☐	☐	☐
12. I enjoy socialising outside of my home environment.	☐	☐	☐	☐	☐
This statement has become less applicable to me in the last 12 months.	☐	☐	☐	☐	☐
13. I would avoid going to a new place if there were a lot of people I didn't know there.	☐	☐	☐	☐	☐
This statement has become more applicable to me in the last 12 months.	☐	☐	☐	☐	☐
14. If a situation arises where I need to speak to someone new, e.g. a new neighbour, I prefer my partner/family member to lead the conversation.	☐	☐	☐	☐	☐
This statement has become more applicable to me in the last 12 months.	☐	☐	☐	☐	☐
15. I am actively involved in my community.	☐	☐	☐	☐	☐
This statement has become less applicable to me in the last 12 months.	☐	☐	☐	☐	☐
16. I think I would find things less stressful if I had more social contact with people.	☐	☐	☐	☐	☐
This statement has become more applicable to me in the last 12 months.	☐	☐	☐	☐	☐
